# Statistical uncertainty quantification to augment clinical decision support: a first implementation in sleep medicine

**DOI:** 10.1038/s41746-021-00515-3

**Published:** 2021-09-30

**Authors:** Dae Y. Kang, Pamela N. DeYoung, Justin Tantiongloc, Todd P. Coleman, Robert L. Owens

**Affiliations:** 1 Department of Medicine, Division of Pulmonary, Critical Care, & Sleep Medicine, University of California, San Diego, 9500 Gilman Dr, La Jolla, CA 92093 USA; 2 Department of Computer Science & Engineering, University of California, San Diego, 9500 Gilman Dr, La Jolla, CA 92093 USA; 3 Department of Bioengineering, University of California, San Diego, 9500 Gilman Dr, La Jolla, CA 92093 USA

**Keywords:** Diagnostic markers, Translational research, Sleep disorders

## Abstract

Machine learning has the potential to change the practice of medicine, particularly in areas that require pattern recognition (e.g. radiology). Although automated classification is unlikely to be perfect, few modern machine learning tools have the ability to assess their own classification confidence to recognize uncertainty that might need human review. Using automated single-channel sleep staging as a first implementation, we demonstrated that uncertainty information (as quantified using Shannon entropy) can be utilized in a “human in the loop” methodology to promote targeted review of uncertain sleep stage classifications on an epoch-by-epoch basis. Across 20 sleep studies, this feedback methodology proved capable of improving scoring agreement with the gold standard over automated scoring alone (average improvement in Cohen’s Kappa of 0.28), in a fraction of the scoring time compared to full manual review (60% reduction). In summary, our uncertainty-based clinician-in-the-loop framework promotes the improvement of medical classification accuracy/confidence in a cost-effective and economically resourceful manner.

## Introduction

The practices of machine learning and artificial intelligence have seen rapid implementation in many facets of today’s society, spanning multiple fields from industrial automation, smart energy and transportation, the internet of things, and medicine^1^. In recent years, there have been many machine learning algorithms for classification and inference leading to automated interpretation of clinical data and generation of decision support tools^2^. As medicine trends towards data-driven practices fueled by aggregation of health and physiologic data points with increased frequency—through the likes of passive monitoring via consumer wearables and initiatives such as the All of Us research program—such analytical methods have become necessary for scalable interpretation and exploration of these data. Recent examples demonstrating the promise of machine learning tools in medicine are Google’s classification of cardiovascular risk from retinal images^3^ and Apple’s watch-based classification of atrial fibrillation^4^. Each of these examples (and many others) seek to characterize and identify clinically relevant adverse health outcomes from stores of data acquired both in and out of the hospital, in an attempt to build a prospective classifier for anticipating human health decline.

Generally, classification algorithms work by utilizing a meaningful subset of raw data as features to best categorize the data into classes of interest. In the case of a probabilistic classifier, a simple way to determine the most appropriate class given the data is to choose the class that maximizes the algorithm’s mathematical argument—the class with the highest likelihood. The result is a classification/label of the data (or estimate of a latent state from which the data were observed) provided in an automated fashion. This basic method of classification can be performed through a variety of machine learning methods—supervised and unsupervised. Though the field of machine learning has progressed in methods of classification, clustering, regression, etc., its measures of success tend to focus on the accuracy of classification: *did the algorithm get the answer correct, possibly compared to some known ground truth*? There exist many methods for assessing the correctness/incorrectness of an algorithm; but when the algorithm is incorrect, in general we do not ascertain *how incorrect* it might be, or how *uncertain* the output was to begin with. The same thinking is useful even when an algorithm is correct compared to the truth—*how correct* was the algorithm during classification? In practice, knowledge of some underlying classification or categorization uncertainty may be useful to better understand how such algorithms work, do not work, and how best to implement their outputs in an interactive framework that would allow for manual review of areas of uncertainty.

Generally, a total measure of uncertainty can be broken down into two components: epistemic uncertainty (that which is knowledge-based, model-driven, systematic, reducible) and aleatoric uncertainty (that which is data-based, statistical, random, or irreducible in nature)^5^. Furthermore, aleatoric uncertainty can be constant (homoscedastic) or vary as a function of the data themselves (heteroscedastic)^6^. The field of uncertainty quantification has seen a proliferation of research characterizing and leveraging these different aspects of uncertainty through novel implementations using Monte Carlo dropout, variational autoencoders, Bayesian neural networks, deep learning ensembles, and uncertainty-aware model architectures^7^. To date, much of this work has been demonstrated on deep learning and reinforcement learning frameworks, with fewer implementations demonstrated using more “traditional” machine learning methods that generally outperform on smaller datasets.

Uncertainty as a metric has been used in many ways—most notably in the form of entropy, a central tenet in information theory^8^ and statistics^9^, with applications as diverse as monitoring infection disease outbreaks^10^, natural language processing^11^, and genomic sequence analysis^12^. Within the context of medicine, pairing uncertainty measures with a label or action has been applied to applications in radiology/nuclear medicine^13^ and more recently in the classification of diabetic retinopathy^14^. Still, the narrative for machine learning in medicine could improve through incorporation of algorithm uncertainty with each clinical estimate. In this sense, algorithmic outputs could have more subtlety. Instead of only a “statement” or firm decision (possibly rivaling the manual equivalent across a large dataset), there could be “estimates” accompanied by a notion of “doubt.” Ultimately, such an approach would serve to add error bars around algorithms and their decisions, allowing clinicians to find confidence in algorithms and enabling a consensus-based approach to medical decision-making. Put another way, such an approach may allow completely automated review of data judged to be of high certainty/low uncertainty, while drawing manual review to areas of low certainty/high uncertainty.

To this end, we describe a novel methodology by which a notion of “uncertainty” can be derived from the conventional outputs of modern probabilistic classifiers and re-incorporated as a mechanism for feedback in decision support. We used automated sleep staging as a toy example of this methodology, wherein epoch-by-epoch sleep architecture was estimated using two machine learning algorithms. Along with sleep stage estimates, we used the algorithms’ conditional posterior probabilities to calculate multiple measures of uncertainty (e.g. posterior variance, Renyi entropy) at each classification epoch. We determined the utility of labeling “uncertain” epochs based on their Shannon entropy, by which uncertain epochs were highlighted to allow targeted clinician-in-the-loop review by an expert and the accuracy of subsequent “clinician + algorithm” results were re-evaluated. The result was a method allowing insight to algorithm performance a priori—without the need to ascertain algorithm correctness to ground truth beforehand—and a mechanism to determine whether an arbitrary classification output should be flagged for further review. If generalized, this method can provide uncertainty measures as a means to inform decision making, classification performance, and online algorithm training in any area of medicine.

Kompa et al.^15^ provide an overview of the advantages to communicating uncertainty when using machine learning methods within the context of medical decision making. In particular, they argue that decision-making tools should have the ability to say “I don’t know” and seek additional human expertise. Such an approach has been used by Abdar et al.^16^ to classify skin lesion as benign, malignant, or “I don’t know”. Similarly, Filos et al.^17^ expand this application to the case of screening for diabetic retinopathy. They also argue not only that uncertainty could be used to determine the need for expert opinion, but that the degree of uncertainty could be used to prioritize those needing more rapid human review. While automated sleep staging has advanced considerably to levels approaching human expert decision making, discrimination between certain sleep stages (e.g. wake and N1) or accuracy for specific patients, especially those with common sleep fragmenting disorders such as obstructive sleep apnea (OSA), remains problematic^18^. Thus, high-throughput sleep analysis requires both automated analysis and the ability to pinpoint studies that require human expert clinician review.

## Results

### Automated scoring and epochs with uncertainty

Subject data such as Apnea-Hypopnea Index, number of epochs/% of total overnight sleep study to be reviewed by clinician, and Cohen’s Kappa are shown in Table 1. For Cohen’s Kappa, four different scoring methods were implemented: (1) initial automated estimate, (2) automated + manual review, (3) automated + clinically relevant review, and (4) automated + substitution.

**Table 1:** Subject data (e.g. AHI), per-subject accuracies, epochs reviewed, scoring time and accuracies.

				Cohen’s Kappa agreement to ground truth			
Subject	AHI (events/h)	# of epochs targeted for review (% of study)	Review time (min)	Automated	Automated + review	Automated + clinically relevant review	Automated + substitution (best)
4	0	222 (20%)	16	0.63	0.66	0.70	0.77
5	1	194 (26%)	33	0.71	0.73	0.77	0.87
15	1	181 (26%)	15	0.62	0.63	0.66	0.80
6	2	276 (25%)	21	0.55	0.54	0.60	0.68
19	2	123 (18%)	16	0.70	0.76	0.79	0.91
3	2	186 (28%)	29	0.67	0.70	0.76	0.85
17	2	389 (22%)	28	0.49	0.48	0.54	0.62
18	3	122 (21%)	18	0.73	0.74	0.79	0.88
20	3	208 (28%)	23	0.64	0.64	0.67	0.80
16	3	173 (38%)	23	0.63	0.71	0.76	0.89
12	7	581 (51%)	31	0.25	0.36	0.40	0.64
13	14	384 (39%)	32	0.38	0.48	0.53	0.74
14	14	507 (53%)	32	0.29	0.35	0.47	0.75
8	19	353 (72%)	30	0.48	0.47	0.67	0.90
2	22	248 (46%)	35	0.63	0.51	0.63	0.93
10	28	312 (45%)	37	0.55	0.57	0.64	0.90
11	33	386 (48%)	33	0.48	0.47	0.53	0.83
9	60	258 (81%)	40	0.55	0.15	0.70	0.95
7	83	278 (49%)	28	0.54	0.46	0.57	0.85
1	94	400 (62%)	28	0.40	0.40	0.56	0.96

**Fig. 1:**
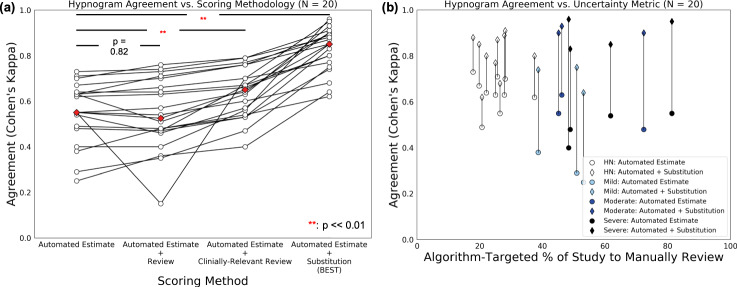
Scoring agreement between ground truth and algorithm. **a** Idealized results for improvement in agreement between ground truth and algorithm estimate + targeted manual review, assuming perfect manual review. **b** Paired agreement values across different hypnograms. Red diamonds = median agreement within each group. Significance determined via paired samples *t* test.

To observe the maximum theoretical possible benefit provided by algorithm-based uncertainty quantification and manual review, we substituted uncertain epochs with the sleep stages in corresponding epochs of the ground truth scoring. In this manner, we assumed that any type of manual review would result in the exact scores provided by the full-PSG scoring, and would indicate the best possible increase in Cohen’s Kappa agreement through scoring intervention via manual review. These automated + substitution results are illustrated in Fig. 1a, for all 20 subjects, stratified by their OSA severity class, along the % of each respective study marked for uncertainty review. We found that perfect correction of uncertain epochs would shift the median **K** value significantly (0.55 → 0.85; *p* = 7.2e−9), as compared to initial automated estimates, and that manual review would provide relatively diminishing returns for decreasing % of study to review. We also found that agreement values trended downward with increases in % of study to review, implying that algorithm uncertainty is associated with overall decreased classification accuracy. Interestingly, using OSA severity stratification, we observed that the studies requiring the least manual review were all HN subjects, and that there is an observable relationship between increased % of study to review and increased OSA severity class.

Surprisingly, manual review did not uniformly result in the expected increase in Cohen’s Kappa agreement, perhaps reflecting uncertainty even between human scorers. Therefore, we restricted review to epochs that were (1) uncertain and (2) had clinical relevance—e.g. the truth was between stage REM vs. N3, as opposed to stage W vs. N1, which is historically difficult to score correctly, even between experts. These automated + clinically relevant review results, alongside the other three scoring methods, are illustrated in Fig. 1b. We observed only mild increases in agreement after raw manual review in some study nights, yielding a decrease in agreement compared to initial automated estimates. After restricting review to those epochs that are uncertain between clinically relevant stages, we observed a statistically significant increase in the group median, compared to the initial automated estimate group, accompanied by a rebound of those study nights that decreased in agreement during raw manual review.

## Discussion

We built a framework allowing specific, targeted intervention by a clinician when an automated algorithm suspects uncertainty in its own classification. The goal of this work was not to build an accurate classifier, per se, but to demonstrate that an imperfect decision support tool (which is often the case) can benefit greatly from the inclusion of uncertainty information. Thus, we can use “clinician-in-the-loop” decision making to improve medical classification accuracy/confidence in a cost-effective and economically resourceful manner. The novelty of our proposed method includes: (1) the use of uncertainty; (2) the quantification of uncertainty derived from the data itself alone, and (3) an example of how this information can be used to minimize clinician time and resources while improving accuracy. Most similar to the methods we describe here, prior work demonstrated entropy quantification in HMM Viterbi sequences^19,20^ showing that uncertainty through entropy could be quantified for the entirety of a latent sequence of states given observed data and model. Later work expanded on this work, showing that local (epoch) uncertainty could be calculated in a computationally efficient manner in Markov processes^21^. Our method is a more direct way to calculate these values on an epoch-by-epoch basis. Although we focus on an example of such “augmented intelligence” in sleep medicine, this method can be applied more broadly in health care.

Overall, the primary advantage of the model described here is the ability to automatically identify epochs of data that present difficulties to the algorithm, which can then be marked for review by an expert. This enables efficient and expedient processing of studies while maintaining the quality required to trust study scoring. Additionally, the model operates directly on the output set of probabilities—which are typical of machine learning classification algorithms—to provide an uncertainty measure for each epoch scored. Thus, this approach can be generalized to any other model designed to classify epochs of data into one of any class labels, so long as the class probabilities add to unity. A disadvantage of the proposed approach is the defined use of the entropy threshold. Certainly, this threshold may vary based on the nature of the problem to be classified or scored, complexity of the data, and extent of model training. Here, the entropy threshold of 1 bit was designed using an understanding of typical sleep stage scoring uncertainty. For other applications, domain expertise will be needed in setting or tuning a threshold value applicable to the model designed and problem at hand. Another limitation is that while the classification algorithm and uncertainty tagging is completely automated, this approach as described requires expert manual review of uncertain epochs. While there are applications where expert perspective is warranted, other settings may require higher throughput (i.e. less manual review) and would need to accept less accuracy. Potentially, complete automation of uncertainty scoring can be accomplished by a simple rule to select the second-most likely class in the uncertain epoch, as indicated by the model, therefore eliminating the need for manual intervention. A more sophisticated approach may be to design a new machine learning model specifically for vetting and re-scoring epochs labeled “uncertain.”

Flagging uncertain epochs was performed by simply thresholding uncertainty values for each epoch in a night’s sleep. The threshold was determined by the value of entropy or variance corresponding to the probabilities of two stages being equally likely, with the remaining three essentially zero-valued. As we have shown, this is an easy way to implement uncertainty quantification and decision support that is also easy to understand. That being said, more sophisticated methods can be employed to provide uncertainty information. For example, Bayesian methods can make use of stage-specific probability distributions of uncertainty alongside prior (expected) information related to the stages and uncertainty values separately. More recently, *dropout* is a method gaining traction for quantifying uncertainty in neural networks, requiring Monte Carlo sampling of the predictive posterior while dropping out neurons from the full network^22^. This methodology is similar, though it simply requires the single calculation of any uncertainty measure discussed from the vector of probabilities at the output during testing.

The metric used to quantify uncertainty, and the cut-off values used, could certainly vary. A variety of other entropy measures that obey basic axioms of uncertainty measures exist^23^. Similarly, whether to review uncertainty from granular data (e.g. epochs) or across an entire data set (e.g. whole night recording) likely depends on the clinical question and need for accuracy. For example, if the clinical question is the presence/absence of obstructive sleep apnea, extremely accurate five-stage classification of sleep may not be needed. Instead, wake vs. sleep or wake vs. non REM vs. REM sleep may be sufficient. Similarly, a precise estimate of the apnea hypopnea index may not be needed, but rather accurate classification of no disease (AHI < 5/h), mild (AHI 5−15/h), or moderate to severe (AHI < 15/h) disease may be sufficient, given both the night to night variability in AHI and that small differences in AHI do not typically affect clinical decision making.

Interestingly, one of the problems we encountered in our approach was the realization that assessment of sleep via PSG is far from a true “gold standard.” Despite the application of a uniform scoring standard, human scorers fail to achieve identical scoring, with classification between certain stages of sleep such as wake vs. N1 particularly troublesome. Our automated scoring system also struggled with these distinctions and uncertainty was often high for these epochs. However, manual review did not uniformly improve scoring agreement with the gold standard since human review is equally fraught with uncertainty. Even big(er)-data approaches fall short of perfect agreement with gold standard criteria. For example, Sun et al.^24^ published work detailing the use of ~1000 features and a feed-forward neural network model for automated sleep staging. As a study employing a plethora of biosignals, the study still only achieved Cohen’s kappa = 0.68 agreement with gold standard manual scoring. Thus, even brute-force approaches to machine learning may not accelerate the accuracy—and therefore complete reliance on—medical classifiers to perfection so quickly. As such, in the interim, use of uncertainty is one method towards augmented clinical scoring and classification. One approach has been by Younes and colleagues who have developed an automated sleep scoring system which can be used to indicate if certain sleep studies require manual editing^25–27^. However, the review rules were not fully automated in that manual review is often based on expectations for a “good” night of sleep, and raw thresholding. Such analysis may miss rare abnormalities of sleep architecture, such as narcolepsy. Future work is needed to investigate the appropriate tolerance level of uncertainty (optimal threshold for flagging/prompting manual review), and applications beyond clinical labeling such as quick screening/diagnosis of obstructive sleep apnea.

Generating and utilizing uncertainty information can be done a priori, that is, without the eyes of manual review. An algorithm can be designed to score sleep architecture in real time, or score a whole night of sleep after the recording is done, and accompany initial estimates and uncertainty of sleep architecture before any manual scoring or review is required. Uncertainty can be assessed at the epoch level, or for a whole night of sleep. The latter may be useful in a rapid hypnogram-based diagnostic tool for sleep apnea, as one expects sleep apnea severity to be correlated with increasingly fragmented sleep, a bane of current automated sleep staging algorithms. Another example of use would pertain to not solely assessing for OSA, but also ruling it out by the use of uncertainty measures that may ultimately lead to an alternative diagnosis such as narcolepsy.

The instantiation of uncertainty quantification here is shown for sleep staging but can be amenable to implementation in other facets of digital medicine necessitating uncertainty measures. All that is required is a set of class probabilities given observations and a model structure, which is the standard output of many modern probabilistic algorithms. Uncertainty-based feedback as outlined seems particularly useful for classification beyond binary classes, since binary classification (healthy vs. disease) can be performed by utilizing the min-entropy, a Renyi entropy pertaining to the negative log of the largest class probability. In practice, using the min-entropy for multi-class classification ignores the probabilities of all other classes (e.g. classes that are not the most likely, but that still may be probable for that specific epoch of classification).

This method of clinician-in-the-loop classification can also be extended to the arena of consensus-based learning and classification, whereby multiple groups tackle a classification problem by offering opinions of a label, each weighted by their specific expertise/knowledge of the task at hand^20^. As originally presented to our clinicians, we included labels for “certain” epochs and did not include the label for “uncertain” epochs—these epochs were presented to clinicians without any label. Our framework could be altered in several ways. For example, the epoch could be labeled with the most probable output, or it could be labeled with the two most likely outputs, e.g. wake vs. N1 sleep. In this latter case, the clinician could determine whether they need to review and decide between these two possibilities, depending on the clinical question. With such a method, the clinician can work more collaboratively with the algorithm. While there is value to developing a more collaborative framework, more research is required to determine the effects/influence of knowing an algorithm’s decision before a clinical decision making.

One the one hand, the abundance of data likely to be generated by wearable technologies will overwhelm existing capabilities of human review. On the other hand, beyond the concept of perfect machine learning classification, practitioners in medicine still maintain the ultimate decision-making power: clinical course is not determined solely by perfect classification, but rather by a number of other factors including such computer-aided analyses, from which clinicians make an informed decision. Clinical procedure is not (and perhaps will never be) at the stage where computers will fully take the wheel—at this time it is prudent to foster tools for enabling clinical decision support, rather than attempt to replace clinician decision making entirely. Our clinician-in-the-loop framework with automated scoring and targeted manual review of areas of uncertainty is one method to balance the data deluge challenge with allowing clinicians to make final decisions. Additionally, this approach provides important context. A step towards combining novel methods of miniaturized sensing (e.g. home sleep test using single-channel EEG) with automated algorithms can enable human-in-the-loop computing to have both the clinician and algorithm collaborate on clinical tasks for improving workflow/operations in a time/cost-efficient manner. Clinicians often weigh the results from different tests (history, physical examination, laboratory, imaging) together to come to a decision. Increasing the input to the clinician from a single test from binary “yes or no”, to “yes, no, or uncertain” may aid in this decision process. Finally, it is impossible to anticipate all clinical scenarios. An ideal clinical support algorithm must perform robustly, and also know when it cannot.

Areas for future work include what level of uncertainty is needed for clinical and research applications. For example, for the majority of clinical practice, accurate determination of wake vs. sleep may be adequate as this determination informs the diagnosis of OSA. In more select cases, such as studies undertaken for the diagnosis of narcolepsy, reliable identification of sleep onset and REM sleep are needed. In this latter case, more human oversight might be needed to confirm the diagnosis. Quantification of uncertainty may better highlight where greater diversity of subjects such as those with more advanced age, those with chronic diseases, or on medications that affect EEG/sleep will be needed as sleep staging becomes increasingly automated in the future, while most algorithms rely on training sets from young and healthy participants.

In summary, measures of uncertainty can be used to target uncertain epochs of algorithm-classified health data for manual review, thereby combining the speed and precision of automated methods with nuanced pattern recognition via manual scoring. Low levels of uncertainty can be used as a decision boundary for a priori screening of (in this case) confident automated sleep staging, indicating nights of sleep requiring more targeted manual scoring attention. A notion of quantified uncertainty in physiologic estimation is a novel method not established. The implementation of such methods could augment future estimation/prediction algorithms, and advance the utility of probabilistic digital medicine.

## Methods

### Participants and data

The study was approved by the UCSD Human Research Protections Program (#160127), and all subjects provided written, informed consent. Data from 40 subjects were gathered from subjects recruited for full in-lab PSG at UC San Diego. Of the 40 study nights, 15 were healthy normals (HN), 8 mild OSA, 8 moderate OSA, and 9 severe OSA. PSG data were recorded using Spike2 software. For each subject, raw single-channel EEG (F3-A2) was derived from full PSG recordings. Single-channel EEG was originally sampled at 250 Hz. Time series EEG data were bandpass filtered between 0.1 and 50 Hz using a zero-phase forward−backward filter (Python, SciPy module). As discussed in a previously published study, time-frequency features of sleep EEG in each epoch of duration 30 s were constructed via multitaper spectrogram, and a non-parametric likelihood model for each of the five sleep stages was constructed via kernel density estimation^28^. Twenty subjects (5 healthy normals, 5 mild, 5 moderate, 5 severe OSA) were used for training the model. Twenty subjects (10 healthy normals, 3 mild, 3 moderate, 4 severe OSA) were used for testing. Also, a multilayer perceptron model neural network classifier (hidden layers of size [32, 64, 32], relu activation, and default parameters as specified in Scikit-learn 0.19.2) was trained and implemented for single-channel automated sleep staging. We employed our data in this algorithm in the same fashion as with the HMM.

After whole-night sleep hypnograms were estimated on a 30-s basis, the a posteriori probabilities of being in any of the five states were calculated for each epoch using the forward−backward algorithm for hidden Markov models. Similarly, softmax probabilities for the neural network were calculated to obtain epoch-specific probabilities of being in any of the five states. With these probabilities, we calculated the variance and Renyi entropy values (for *α* = 1, 2, and infinity, corresponding to Shannon entropy, collision entropy, and min entropy, respectively) of the probability distributions in each 30-s epoch. For an epoch with probabilities *p* = [*p*_1_,…*p*_5_], the Reny entropy is given by Eq. (1):

1 $$S_\alpha \left( p \right) = \frac{1}{{1 - \alpha }}\log _2\left( {\mathop {\sum}\limits_{i = 1}^5 {p_i^\alpha } } \right)$$

with *α* = 1 being the Shannon entropy as a special case in Eq. (2):

2 $$S\left( p \right) = \left( {\mathop {\sum}\limits_{i = 1}^5 { - p_i\log _2p_i} } \right)$$

This results in a “time series” of uncertainty values related to the algorithms’ estimate of the sleep stage at each corresponding epoch. Since the number of states is *N* = 5 for the sleep staging problem, the per-epoch value of Shannon entropy *S* lies between 0 (absolute certainty) to 2.32 bits (absolute uncertainty). We specified the threshold of uncertainty at *S*_threshold_ = 1 bit, pertaining to a scenario where two of the possible states are equally likely and all other states are unlikely to occur (e.g. *p* = [~0, ~0, ~0, ~0.5, ~0.5]). For any epoch where *S*_epoch_ > *S*_threshold_, the epoch was marked for targeted manual review by a Registered PSG Technician (RPSGT) who was blinded to the initial manual or automated scoring. The time needed for manual review was recorded by the technician.

### Implementation details

#### A clinician-in-the-loop workflow—quantifying epoch-by-epoch algorithm uncertainty in sleep staging

**Fig. 2:**
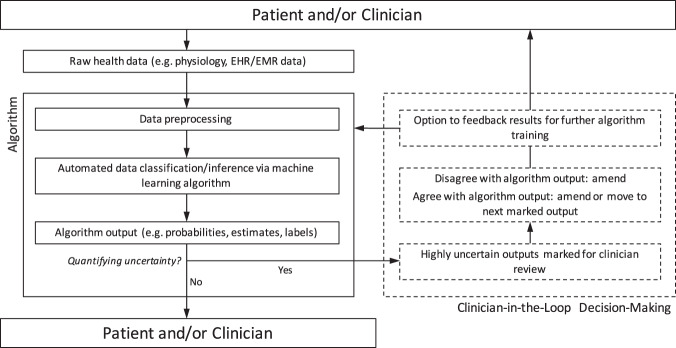
Generalized workflow diagram for clinician (human) in-the-loop classification. With measures of uncertainty, data can be reviewed by expert clinicians or used for further algorithm training.

Figure 2 illustrates a set of conceptual frameworks using a machine learning algorithm for medical classification followed by an option to quantify uncertainty in the algorithm output. If uncertainty is not quantified, the algorithm output is returned to the patient and/or clinician without indication of performance. Conversely, if uncertainty is quantified, highly uncertain outputs can be marked or tagged for manual review by a clinician. This pathway allows for a resource-efficient, targeted review of specific outputs or data, as opposed to brute-force, exhaustive review of all algorithm outputs. In this manner, a clinician has the opportunity to agree with the algorithm if he/she feels it is correct, or the clinician can disagree with the algorithm and amend the output. From the lens of uncertainty quantification, the former is somewhat of a false-negative as the algorithm marked the output as highly uncertain, but was in fact correct, while the latter represents a true-negative in that the algorithm correctly identified the output as uncertain, allowing for correction at the level of manual review. After review, there is an opportunity to feedback these corrected results into the algorithm for further supervised learning, by which the algorithm training and classification process can continue in a clinician-informed manner.

**Fig. 3:**
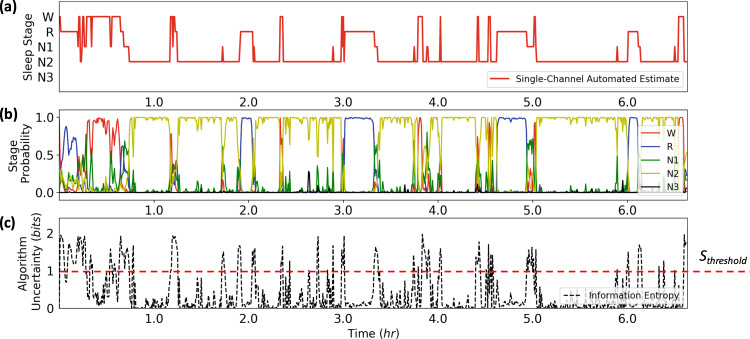
Generation of hypnogram with underlying uncertainty. **a** Automated hypnogram estimate for an HN subject. **b** Probabilities determining the estimated sleep stages. **c** Raw algorithm uncertainty values before thresholding. *S*_threshold_ pertains to the Shannon entropy cutoff (1 bit).

The algorithm employed in this work provided single-channel automated estimates of overnight sleep architecture (Fig. 3a). Underlying the epoch-by-epoch classification of sleep stages in the algorithm is a set of probabilities pertaining to the conditional likelihood of a sleep stage given the data and algorithm model (Fig. 3b). The value of *S*_epoch_ was calculated for each epoch and subject to a threshold *S*_threshold_ = 1 bit to generate the labels for targeted review by an RPSGT.

#### A deconstructed class confusion matrix and class distributions of per-epoch Shannon entropy

To assess the accuracy of uncertainty classification, we created a confusion matrix illustrating the percentage of all epochs in which the algorithm was certain (*S*_epoch_ < *S*_threshold_) about its sleep stage estimate (Table 2). We observed that of those epochs scored with certainty, stages W (87%) and N2 (90%) were most accurately classified compared to the ground truth. For the remaining three stages (REM, N1, and N3), we found the algorithm estimated these epochs with certainty, but fell short in accuracy (73, 60, and 65%, respectively). However, these numbers are comparatively good considering the task of single-channel automated sleep scoring and the fact that these stages are often the root of discrepancies in inter-rater agreement^29–31^.

**Table 2:** Certain sleep stage classification confusion matrix.

	Wake	REM	N1	N2	N3	True stage
Wake	**87%**	1	8	4	0	
REM	5	**73%**	10	12	0	
N1	9	1	**60%**	30	0	
N2	0	1	5	**90%**	4	
N3	0	1	0	34	**65%**	
Certain estimate						

Values in bold represent uncertainty algorithm true-positive rate.

Similarly and conversely, we analyzed the percentage of all epochs for which the algorithm was uncertain (*S*_epoch_ > *S*_threshold_) (Table 3). We observed that the algorithm seemed most uncertain about epochs that are incorrectly scored as stages W, N1, and N2 when they are most often stages N1, N2, and N2, respectively. In a sense, the diagonal of the uncertain confusion matrix can be considered as the false-negative rate—the algorithm marked these epoch estimates as uncertain, but actually correctly classified the sleep stage when compared to the ground truth. Another way to interpret this uncertain matrix is that the algorithm correctly marked uncertain estimates in REM and N3 staging that would lead to a different stage 75 and 76% of the time, respectively. Viewing the data this way, the off-diagonal values of the uncertain matrix represent second-chance opportunities for correct classification available during manual (or further automated review) of algorithm-marked uncertain epochs.

**Table 3:** Uncertain sleep stage classification confusion matrix.

	Wake	REM	N1	N2	N3	True stage
Wake	**44%**	5	34	16	1	
REM	15	**25%**	27	33	0	
N1	10	7	**51%**	32	0	
N2	2	5	19	**70%**	4	
N3	1	5	3	67	**24%**	
Uncertain estimate						

Values in bold represent false-negative rate.

**Fig. 4:**
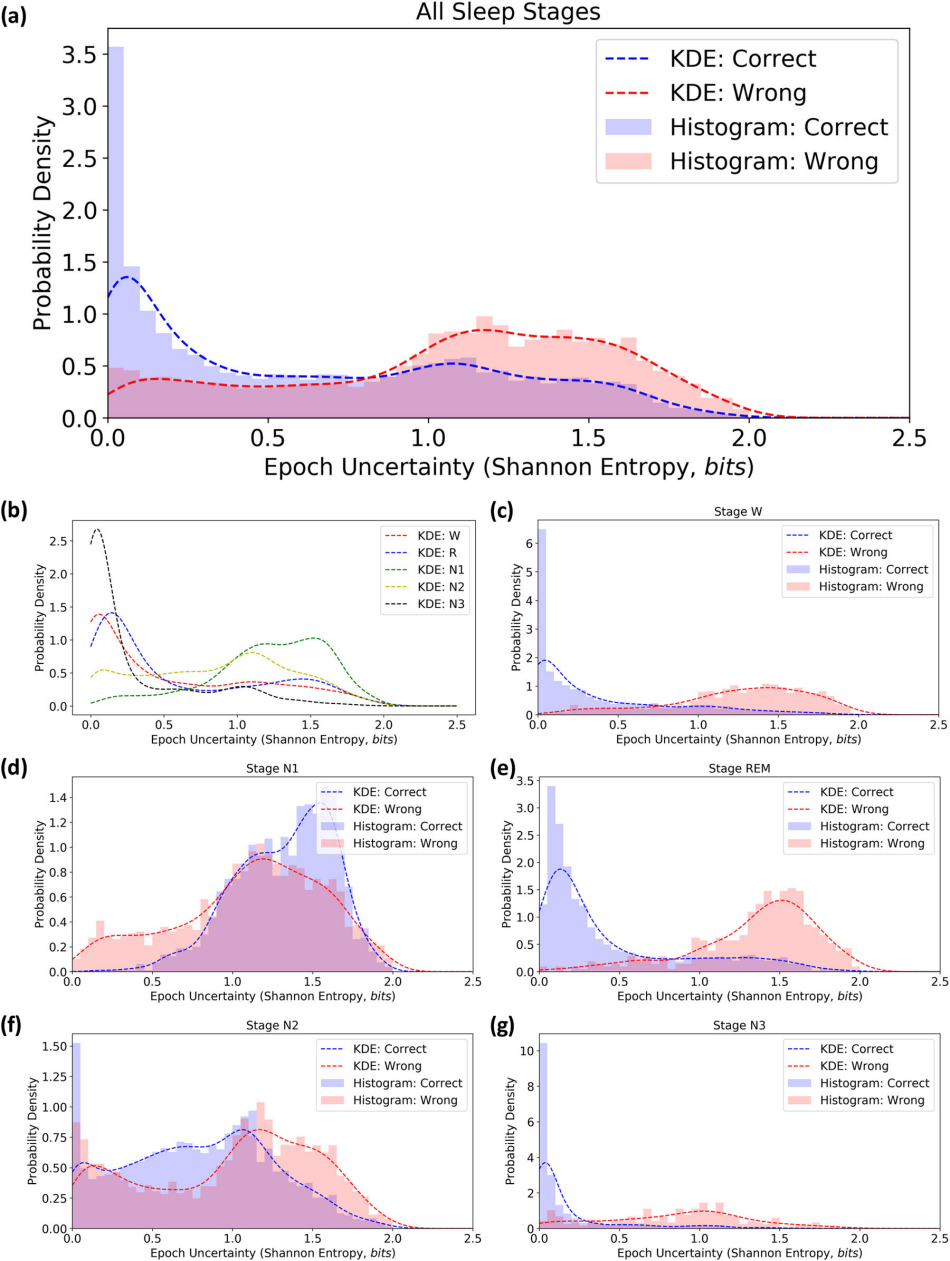
Entropy distribution plots for all and per-stage distributions using HMM-based classification. **a** Shannon entropy distribution + correctness for all stages. **b** Shannon entropy distribution per class. Shannon entropy distribution and correctness for stage: **c** W (3602 epochs), **d** REM (2320 epochs), **e** N1 (3222 epochs), **f** N2 (6653 epochs), and **g** N3 (1629 epochs).

To further understand algorithm uncertainty quantification, we generated histograms and probability distribution functions of the per-epoch Shannon entropy using all 17,426 epochs. Figure 4a illustrates the distribution of entropy values for both correct (11,645 epochs) and incorrect (5781 epochs) epochs, along with corresponding kernel density estimation (KDE) fits. We observed that epochs correctly classified demonstrated a left-leaning distribution and lower overall values of entropy (0.63 ± 0.55 bits), compared to those incorrectly classified (1.07 ± 0.51 bits) which appeared to be more evenly distributed along the domain of possible entropy values. Stratifying these results further into distributions for specific sleep stages (Fig. 4b−g), we found that stages W, REM, and N3 demonstrated similar distribution features—correct epochs with entropy values close to 0 bits (W: 2899 epochs, 0.38 ± 0.46 bits; REM: 1901 epochs, 0.44 ± 0.47 bits; N3: 1395 epochs, 0.18 ± 0.31 bits) and incorrect epochs extending outward toward and past 1 bit of entropy (W: 703 epochs, 1.25 ± 0.44 bits; REM: 419 epochs, 1.35 ± 0.39 bits; N3: 234 epochs, 0.85 ± 0.44 bits). Interestingly, stages N1 and N2 (Fig. 4e, f) appeared to exhibit the most overlap in correct distributions (N1: 1609 epochs, 1.32 ± 0.30 bits; N2: 3841 epochs, 0.79 ± 0.47 bits) vs. incorrect distribution (N1: 1613 epochs, 1.11 ± 0.46 bits; N2: 2812 epochs, 0.98 ± 0.54 bits). This implies that the algorithm’s certainty in its own estimate for these stages is much less clear and could possibly lead to an increase in false targeting and/or decreased true targeting for manual review, relative to the aforementioned stages.

#### Enabling targeted manual review of uncertain epochs

Using the uncertainty framework described, we sought to assess the utility of clinician-in-the-loop manual review of targeted epochs. Each of the 20 test study nights were automatically scored and assessed by the algorithm for single-channel sleep stage estimates and uncertainty. In each study night, uncertain epochs in the initial automated estimates were targeted for manual review by an RPSGT, while the remainder of the (certain) automated estimates were unmodified. Specifically, the RPSGT was allowed to observe the uncertain epochs along with all PSG channels (e.g. flow, EEG, oximetry) before, during, and after the targeted epoch to arrive at a review decision: agree with the algorithm’s estimate, or change it to one of the other four sleep stages. After manual review of all uncertain epochs, these combined automated + manual review estimates and the initial automated estimates were evaluated for agreement against the ground truth via Cohen’s Kappa (**K**)^32^.

**Fig. 5:**
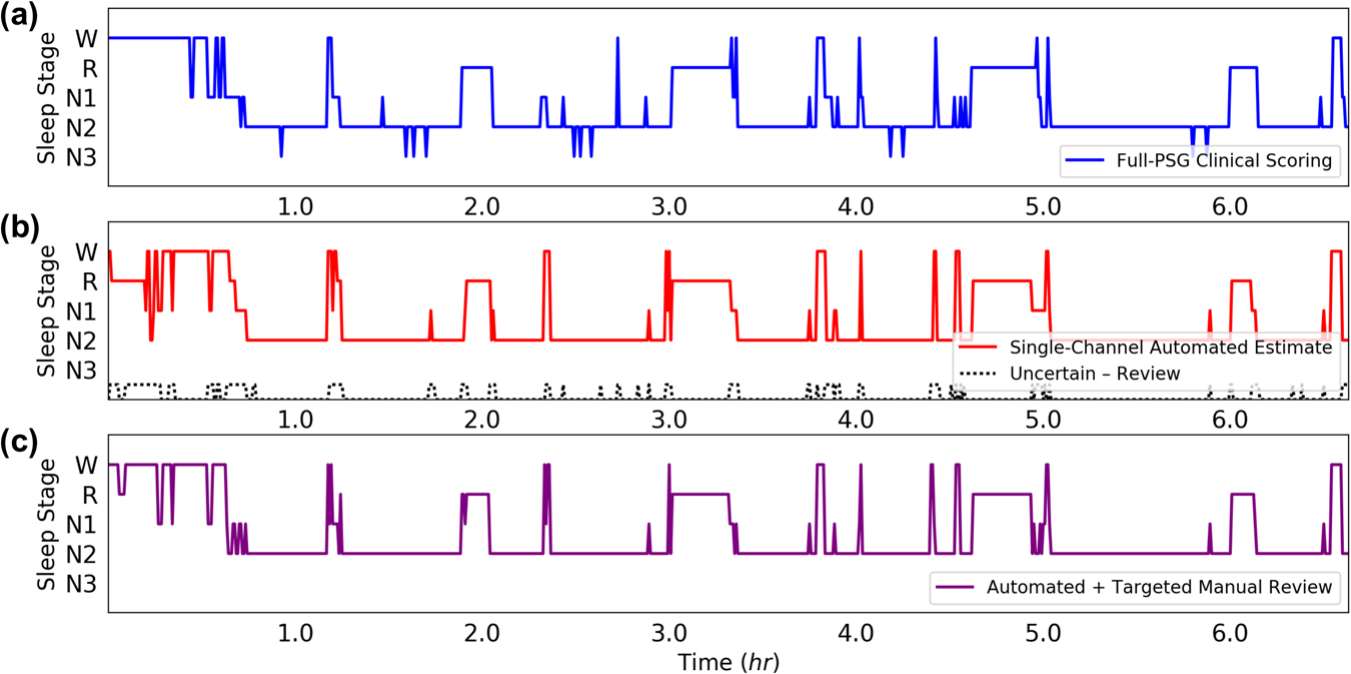
Hypnogram generation for an individual. **a** Hypnogram based on full-PSG and manual scoring for an HN subject (AHI < 5). **b** Automated hypnogram and epochs threshold and targeted for manual review. **K** = 0.70, 18% of study to review. **c** Automated hypnogram after manual review of uncertain epochs. **K** = 0.76.

An example of these data in an HN subject is illustrated in Fig. 5. Each study had full-PSG scoring (Fig. 5a), which was used as ground truth in comparison to initial automated estimates (Fig. 5b). Uncertain epochs targeted for manual review were indicated by a binary sequence of 1’s and 0’s—where 1’s represent “uncertain” and 0’s represent “certain” epochs after thresholding by *S*_epoch_ values by *S*_threshold_ = 1 bit. Manual review of only the uncertain epochs resulted in automated + manual review estimates (Fig. 5c). In this specific study night, the initial automated estimate had a **K** = 0.70 (substantial agreement) when compared to ground truth. After a 16-min manual review of only 18% of the study (123 epochs), Cohen’s Kappa increased to **K** = 0.76. During manual review, we observed that the reviewer was able to rectify algorithm misclassification of stage REM to stage W during uncertain epochs in the first hour of the study. Similarly, the reviewer was able to correct misclassification of stage N1 to stage N2 just shy of hours four and five of the study.

### Reporting summary

Further information on research design is available in the Nature Research Reporting Summary linked to this article.

## Supplementary information

Reporting Summary

## Data Availability

The datasets generated during and/or analyzed during the current study are available from the corresponding author on reasonable request.

## Code availability

The code may be made available from the authors upon reasonable request subject to permission and approval from the corresponding organizations and institutional review boards.
