# Supplementary material for: Statistical uncertainty quantification to augment clinical decision support: a first implementation in sleep medicine
Source: NPJ Digit Med. 2021 Sep 30;4:142. doi: 10.1038/s41746-021-00515-3 (PMC8484290; doi:10.1038/s41746-021-00515-3)
Supplement: Supplementary file 1 — Reporting Summary [file 41746_2021_515_MOESM1_ESM.pdf]

nature portfolio

Corresponding author(s): Robert L. Owens, MD
Last updated by author(s): Sep 3, 2021

Reporting Summary

Nature Portfolio wishes to improve the reproducibility of the work that we publish. This form provides structure for consistency and transparency in reporting. For further information on Nature Portfolio policies, see our Editorial Policies and the Editorial Policy Checklist.

Statistics

For all statistical analyses, confirm that the following items are present in the figure legend, table legend, main text, or Methods section.

n/a	Confirmed	
☐	☒	The exact sample size (*n*) for each experimental group/condition, given as a discrete number and unit of measurement
☐	☒	A statement on whether measurements were taken from distinct samples or whether the same sample was measured repeatedly
☐	☒	The statistical test(s) used AND whether they are one- or two-sided<br>*Only common tests should be described solely by name; describe more complex techniques in the Methods section.*
☒	☐	A description of all covariates tested
☒	☐	A description of any assumptions or corrections, such as tests of normality and adjustment for multiple comparisons
☐	☒	A full description of the statistical parameters including central tendency (e.g. means) or other basic estimates (e.g. regression coefficient) AND variation (e.g. standard deviation) or associated estimates of uncertainty (e.g. confidence intervals)
☐	☒	For null hypothesis testing, the test statistic (e.g. *F*, *t*, *r*) with confidence intervals, effect sizes, degrees of freedom and *P* value noted<br>*Give P values as exact values whenever suitable.*
☒	☐	For Bayesian analysis, information on the choice of priors and Markov chain Monte Carlo settings
☒	☐	For hierarchical and complex designs, identification of the appropriate level for tests and full reporting of outcomes
☒	☐	Estimates of effect sizes (e.g. Cohen's *d*, Pearson's *r*), indicating how they were calculated

Our web collection on statistics for biologists contains articles on many of the points above.

Software and code

Policy information about availability of computer code

Data collection	Sleep study variables collected using Spike2 software, Cambridge Electronic Design Limited, Cambridge, UK
Data analysis	Python, SciPy module available from SciPy.org

For manuscripts utilizing custom algorithms or software that are central to the research but not yet described in published literature, software must be made available to editors and reviewers. We strongly encourage code deposition in a community repository (e.g. GitHub). See the Nature Portfolio guidelines for submitting code & software for further information.

Data

Policy information about availability of data

All manuscripts must include a data availability statement. This statement should provide the following information, where applicable:

- Accession codes, unique identifiers, or web links for publicly available datasets
- A description of any restrictions on data availability
- For clinical datasets or third party data, please ensure that the statement adheres to our policy

The datasets generated during and/or analysed during the current study are available from the corresponding author on reasonable request.

Field-specific reporting

Please select the one below that is the best fit for your research. If you are not sure, read the appropriate sections before making your selection.

☒ Life sciences ☐ Behavioural & social sciences ☐ Ecological, evolutionary & environmental sciences

For a reference copy of the document with all sections, see nature.com/documents/nr-reporting-summary-flat.pdf

Life sciences study design

All studies must disclose on these points even when the disclosure is negative.

Sample size	Convenience sample of 40 subjects (20 training, 20 validation), in line with prior similar studies.
Data exclusions	None
Replication	This study is a proof of concept and there was no effort made to replicate the findings in another dataset.
Randomization	Data were obtained from patients with and without obstructive sleep apnea. The training and validation groups were chosen to have roughly equal patients with no, mild, moderate and severe obstructive sleep apnea.
Blinding	Scoring of all sleep studies was done in blinded fashion.

Reporting for specific materials, systems and methods

We require information from authors about some types of materials, experimental systems and methods used in many studies. Here, indicate whether each material, system or method listed is relevant to your study. If you are not sure if a list item applies to your research, read the appropriate section before selecting a response.

Materials & experimental systems

n/a	Involved in the study	
☒	☐	Antibodies
☒	☐	Eukaryotic cell lines
☒	☐	Palaeontology and archaeology
☒	☐	Animals and other organisms
☐	☒	Human research participants
☐	☒	Clinical data
☒	☐	Dual use research of concern

Methods

n/a	Involved in the study	
☒	☐	ChIP-seq
☒	☐	Flow cytometry
☒	☐	MRI-based neuroimaging

Human research participants

Policy information about studies involving human research participants

Population characteristics	Age>18 years, without and without obstructive sleep apnea, and free from other chronic medical conditions. Also free from medications.
Recruitment	Subjects were recruited via community advertisements and word of mouth from the community as well as the Sleep Medicine clinic
Ethics oversight	University of California San Diego Human Research Protections Program.

Note that full information on the approval of the study protocol must also be provided in the manuscript.

Clinical data

Policy information about clinical studies

All manuscripts should comply with the ICMJE guidelines for publication of clinical research and a completed CONSORT checklist must be included with all submissions.

Clinical trial registration	This was not a clinical trial
Study protocol	The study protocol is available upon reasonable request to the corresponding author.
Data collection	Data were acquired in the USCD Sleep Medicine Research Laboratory from January 2016 through December 2018

Outcomes	Main outcome in the present study was time needed for manual review of each sleep study. This time for each study was recorded by the registered polysomnographic technician.
